# Spatial modelling of type II diabetes outcomes: a systematic review of approaches used

**DOI:** 10.1098/rsos.140460

**Published:** 2015-06-17

**Authors:** Jannah Baker, Nicole White, Kerrie Mengersen

**Affiliations:** 1 School of Mathematical Sciences, Queensland University of Technology, Brisbane, Queensland, Australia; 2 Cooperative Research Centres for Spatial Information, Melbourne, Victoria, Australia

**Keywords:** diabetes, spatial, geographic, mapping, systematic review

## Abstract

With the rising incidence of type II diabetes mellitus (DM II) worldwide, methods to identify high-risk geographical areas have become increasingly important. In this comprehensive review following Cochrane Collaboration guidelines, we outline spatial methods, outcomes and covariates used in all spatial studies involving outcomes of DM II. A total of 1894 potentially relevant citations were identified. Studies were included if spatial methods were used to explore outcomes of DM II or type I and 2 diabetes combined. Descriptive tables were used to summarize information from included studies. Ten spatial studies conducted in the USA, UK and Europe met selection criteria. Three studies used Bayesian generalized linear mixed modelling (GLMM), three used classic generalized linear modelling, one used classic GLMM, two used geographic information systems mapping tools and one compared case:provider ratios across regions. Spatial studies have been effective in identifying high-risk areas and spatial factors associated with DM II outcomes in the USA, UK and Europe, and would be useful in other parts of the world for allocation of additional services to detect and manage DM II early.

## 1. Background

Type II diabetes mellitus (DM II) is on the rise worldwide and is reported to be increasing in every country of the world [1]. Costly sequelae of DM II, including hospital admission, can be prevented with quality primary care [2]; however, many areas suffer from insufficient primary care services to meet the growing epidemic of DM II. Indeed, diabetes care has been reported to be ‘in a state of crisis’ in a recent report from Diabetes UK [3]. An estimated 50% of DM II cases are undiagnosed [1]. Spatial studies that identify high-risk areas for DM II outcomes can highlight regions that would most benefit from additional primary care services to detect, manage and monitor DM II early. In addition, spatial studies have the potential to identify geographical factors that are important to DM II aetiology. We define spatial studies as studies involving aggregate or point-level spatial information. This broad definition includes ecological and multi-level studies, and models with correlated and uncorrelated spatial effects.

A spatial approach to disease modelling is relevant to any chronic disease with elements of environmental causation and is especially relevant to DM II as recent research has demonstrated associations between DM II prevalence and geographical factors such as green space, walkability, increased fast-food availability, car-dominated transport and reduced opportunities for exercise [4,5]. Many of these factors are amenable to health promotion programmes, and spatial analysis can provide useful information to inform resource allocation and public policy decisions [6]. As yet, there are few spatial studies in the literature examining DM II outcomes, and a review of the findings and methodologies used in these studies would be useful as a basis for further studies in other parts of the world to identify areas in need for DM II management, and associated geographical factors amenable to modification.

Currently, 11.3% of the USA and 4.45% of the UK adult population are estimated to have diabetes, and DM II accounts for 90–95% of these cases [7,8]. Diabetes is the leading cause of renal failure, non-traumatic lower-limb amputation, and new cases of blindness, the major cause of heart disease and stroke, and the seventh leading cause of death in the USA [7]. The direct and indirect costs of diabetes are estimated to exceed USD 612 billion in the USA in 2014, £23.7 billion in the UK in 2011 and AUD 14.6 billion in Australia in 2010 [9–11].

The management of DM II is complex and time-consuming and may involve regular health consultations, lifestyle modification, frequent blood glucose and podiatry checks and complex medication regimes [12]. Fortunately, there is evidence that around 60% of DM II cases are preventable with lifestyle change and/or medications [13]. Early detection and management of glycaemic control and cardiovascular risk factors should lead to more effective treatment while reducing the risk of diabetic complications [14]. Screening for undiagnosed cases using a fasting plasma glucose test thus has the potential to significantly reduce the healthcare burden of DM II [14]. Effective placement of screening services can be determined using spatial analysis of DM II outcomes to identify areas of high risk.

Numerous studies, including a systematic review, suggest that a number of demographic and clinical factors and metabolic markers are associated with increased risk of developing DM II [15–19]. In addition, there is some evidence of geographical factors associated with DM II prevalence [4,5,17]. These are summarized in table 1. Several of these factors are modifiable, including lifestyle choices and associated cardiovascular risk, and neighbourhood factors are amenable to health promotion programmes. Owing to spatial clustering of lifestyle factors, identification of areas with higher prevalence of lifestyle-related risk factors would allow provision of targeted health promotion programmes.

**Table 1.** Risk factors associated with increased risk of developing type II diabetes mellitus.

demographic factors	metabolic markers
male gender	elevated fasting plasma glucose
increasing age	elevated 2-h post-prandial glucose
increasing BMI	elevated random glucose
indicators of low socio-economic status (education, income, occupation)	elevated triglyceride:high-density lipoprotein ratio
increasing waist:hip ratio	white cell count
increasing waist:height ratio	elevated HbA1c
black/hispanic ethnicity	elevated interleukin-2 receptor A
sedentary lifestyle/physical inactivity	elevated adiponectin
smoking history	elevated C-reactive protein
excessive alcohol use	elevated ferritin
low levels of fruit and vegetable consumption	elevated Ga-glutamyl transpeptidase
—	elevated insulin level

clinical factors	environmental factors
hypertension	reduced green space/walkability
cardiovascular disease	increased fast-food availability
tachycardia	decreased access to healthy food
family history of diabetes in first degree relative	car-dominated transport
history of gestational diabetes	reduced opportunities for exercise
corticosteroid use	lower SES
—	higher proportion of daily smokers

Diagnosis of DM II appears to be associated with diagnosis of several other disorders, including hypertension [20], coronary arterial disease [21–23], congestive heart failure [24,25], chronic obstructive pulmonary disease [26,27], colorectal cancer [28–31], pancreatic cancer [32–34], endometrial cancer [35], acute pancreatitis [36], biliary disease [36], psoriasis [37], urinary tract calculi [38] and diagnosis with high-grade prostate cancer [39]. Spatial analyses allow examination of joint spatial correlations between multiple diseases, and describing these methodologies would be useful for future research into geographical associations between these diseases.

Historically, geographical studies have been effective in finding associations between incidence and mortality of disease and exposure to risk factors, such as lifestyle and environmental factors [40]. Software packages, such as BUGS, R, MapInfo are available for performing spatial mapping of disease outcomes and their relationship with exposure to lifestyle and environmental factors [41–43]. Based on a small range of standard algorithms, these software packages provide smoothed estimates and colour-coded geographical maps. Maps provide a powerful visual tool for identification of geographical patterns of occurrence of disease and are potentially useful in the formulation of hypotheses of DM II aetiology.

Both purely ecological and multilevel studies are useful for ascertaining risk factors underlying spatial variation in DM II outcomes. Purely ecological studies using aggregate spatial data have the advantage that they avoid ethical and confidentiality considerations associated with the identifiability of individuals. Survey data for DM II outcomes and geographical factors aggregated to small area level are readily available, and by aggregating measurements over multiple persons, data may be associated with less measurement error than individual data [44]. Ecological studies are thus able to identify associations between disease outcomes and geographical factors at a small area level.

By contrast, multilevel studies that account for individual-level factors nested within geographical factors have the advantage of being able to identify the residual effects of geographical factors on DM II outcomes after accounting for individual factors, and to measure the relative importance of each. However, obtaining data at both an individual and geographical level requires more effort and ethical consideration.

Bayesian models are particularly well suited to spatial modelling as the information specific to each region can naturally be represented as priors, and both correlated and uncorrelated spatial effects can be examined [45]. The Bayesian framework accounts for different sources of uncertainty and compensates for sparse and missing data. For sparse data, incorporation of spatially correlated priors for residual error allows ‘borrowing of strength’ across neighbouring regions allowing for more robust inferences. Use of spatially correlated priors can also be a good method for imputation of missing outcome or covariate information [46]. In addition, uncertainty around notification rates and measurement error can be incorporated into a Bayesian model. Furthermore, hierarchical Bayesian models allow the exploration of individual-level risk factors nested within correlated and uncorrelated spatial effects.

DM I and DM II differ in underlying aetiological factors; however, many spatial studies analyse both in combination. As the vast majority of diabetic cases (90–95%) are accounted for by DM II, inclusion of these studies is still useful for examination of DM II outcomes, as the remaining small proportion of DM I cases is unlikely to bias results very much. Thus, inferences from spatial studies combining DM I and DM II are also useful for the purpose of this review.

This systematic review aims to perform a comprehensive search of the literature in accordance with Cochrane Collaboration guidelines to identify all spatial studies available involving aggregate or point-level spatial information, and examining outcomes of DM II, or DM I and DM II combined. This review aims to summarize: (i) risk factors for DM II identified by spatial studies, (ii) general spatial methods used, and (iii) to describe in detail statistical analyses used in these studies.

## 2. Material and methods

### 2.1 Search strategy

A systematic review was conducted to identify all articles published between January 1950 and June 2013 involving spatial methodology to examine outcomes of DM II, or DM I and DM II combined. The eight steps of the Cochrane Collaboration guidelines for a systematic review below were followed [47]:

step 1: defining the review questions and developing criteria for including studies;
step 2: searching for studies;
step 3: selecting studies and collecting data;
step 4: assessing risk of bias in included studies;
step 5: analysing data and undertaking meta-analyses;
step 6: addressing reporting biases;
step 7: presenting results and ‘summary of findings’ tables; and
step 8: interpreting results and drawing conclusions.

The review questions (step 1) were:

— what health outcomes and covariates have been examined in spatial studies involving outcomes of type II diabetes mellitus?
— what spatial methods have been used in these studies?

Systematic searches (step 2) were performed using MEDLINE, Science Direct, Web of Science, CINAHL and Cochrane Library of Systematic Reviews. A combination of Medical Subject Headings (MeSH) and keyword searches were used. For MEDLINE, which allowed the most tailored search strategy, two subsets of citations were generated, to identify studies using spatial or Bayesian methodology. The MeSH term ‘diabetes mellitus type 2’ was used for participant type, combined with (i) keywords ‘map*’, ‘geographic’, ‘spatial’, ‘areal’ or ‘belt’ to identify spatial studies and (ii) truncated keyword ‘bayes*’ to identify Bayesian studies (* indicates truncation). Searches were limited to English literature and human studies. The search terms ‘type 2 diabetes mellitus’ combined with ‘map*’, ‘geographic’, ‘spatial’, ‘areal’ or ‘belt’ were also used to search Science Direct, Web of Science and CINAHL. Searches were restricted to abstract, title or keywords for Science Direct, topics for Web of Science and abstract for CINAHL. The Cochrane Library of Systematic Reviews was searched using the term ‘type 2 diabetes mellitus’. Reference lists of relevant articles identified by this method were scanned for other studies not identified through the electronic search.

### 2.2 Selection of studies

**Figure 1.**
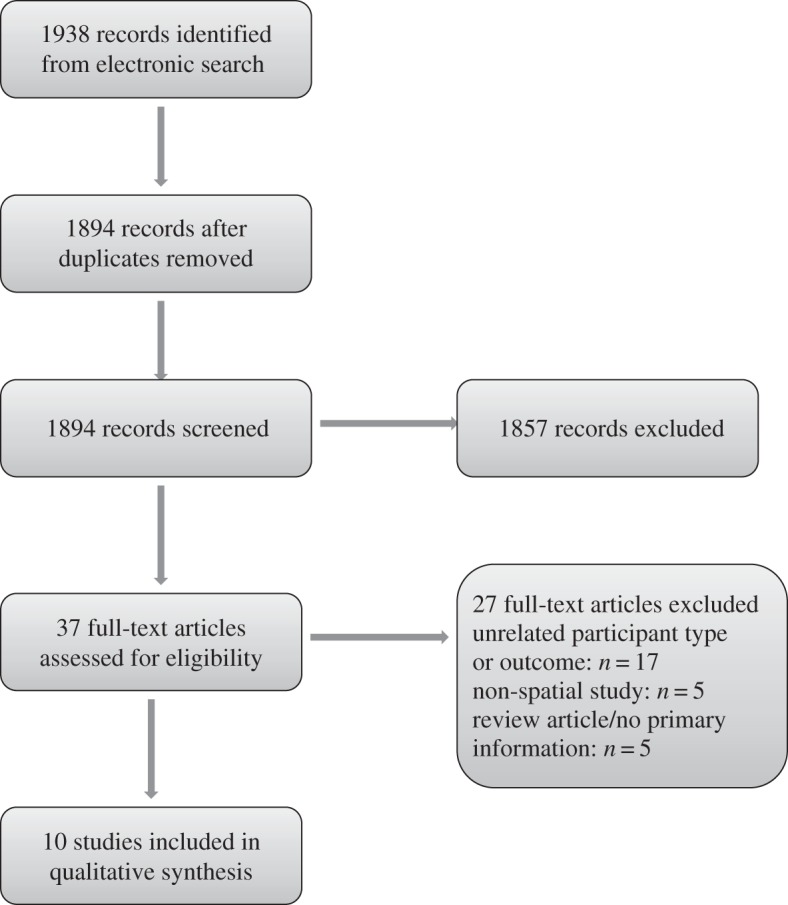
Study selection and exclusion process.

Predefined inclusion and exclusion criteria were set for study selection prior to conducting the literature search (step 3). Studies were included if they used spatial methods to explore aggregate or point-level spatial data examining outcomes of DM II, or DM I and DM II combined, and excluded if only non-spatial methods were used or only involved participants with DM I. A two-stage process was used to select relevant studies for the review (figure 1). One author (J.B.) independently examined abstracts of all articles identified through electronic searches and excluded those not meeting the selection criteria. Following this, three authors (J.B., N.W. and K.M.) independently examined full manuscripts obtained and made decisions about inclusion and exclusion of studies. Any disagreement was resolved through discussion. Where more than one article was found describing the same study, only the most recent or most complete publication was included unless methodology used differed significantly.

### 2.3 Data extraction

Data extraction was performed by one author (J.B.) and entered into a predesigned spreadsheet. For each included study, information was extracted about geographical location, sample size, outcome measures examined, statistical methods used, covariates included and results found. Characteristics and results of each study are summarized into a table.

## 3. Results

The electronic search identified a total of 1938 potentially relevant citations from all databases searched, and after removal of 44 duplicates, abstracts of 1894 citations were screened. Overall, 1857 citations were excluded on the basis of abstract information (figure 1). Full manuscripts were obtained and examined for 37 articles, and 10 studies met selection criteria, of which three used Bayesian spatial methodology and seven used classical modelling techniques. Risk of bias was assessed (step 4), and coverage bias was a possibility with all studies included but difficult to assess. Other types of bias described in the Cochrane Handbook, including selection, performance, detection, attrition and reporting bias, relevant to studies comparing two arms, were less relevant to the spatial studies included.

A descriptive analysis was performed (step 5). Owing to large differences in outcomes measured and methodology used between studies, meta-analysis was not possible, nor was publication bias able to be assessed via funnel plots and sensitivity analyses (step 6). Table 2 outlines characteristics of studies included in this systematic review, including details of study location, sample size, outcome measures, methodology used, covariates included and results found (step 7).

**Table 2.** Characteristics of included studies. (US, United States; b/w, between; DM, diabetes mellitus; DM I, type I diabetes mellitus; DM II, type II diabetes mellitus; CAR, conditional autoregressive; MCAR, multivariate conditional autoregressive; HbA1c, glycated haemoglobin; GIS, geographical information science; SES, socio-economic status; +ve, positive; hx, history; CVD, cardiovascular disease; HT, hypertension.)

primary author, year	country	sample size	outcome measures	methods	covariates in model	results
Liese 2010 [48]	US	four US regions	geographical variation, joint spatial correlation b/w DM I and DM II, smoothed risk estimates	sparse Poisson CAR, MCAR	age, gender, ethnicity	evidence for small area variation in incidence of and joint correlation between DM I and DM II
Geraghty 2010 [49]	US	7288 DM (I or II) pts	DM prevalence, distance to primary care provider, glycaemic control (HbA1c)	regression, geographical information software (GIS) mapping	demographic and laboratory characteristics	SES barrier to optimal glycaemic control
Lee 2008 [50]	US	nine US regions	disparities between estimated paediatric DM prevalence and endocrinologist supply	mapping of DM prevalence:paediatric endocrinologist ratio	—	up to 19-fold difference in case:provider ratio across regions
Green 2003 [17]	US	230 Manitoba areas	DM prevalence estimation	spatial scan statistic, spatial autoregressive linear regression	sociodemographic, environmental and lifestyle factors	low SES, poor environmental quality, poor lifestyles +ve correlated with DM prevalence
Noble 2012 [51]	England	130 areas in London	small-area mapping of 10-year risk of developing DM II	GIS mapping	age, gender, ethnicity, deprivation, family hx, CVD, smoking, HT, steroid use, height, weight	small-area geospatial mapping feasible
Congdon 2006 [52]	England	8000 electoral wards	DM prevalence estimation	regression, aggregate data	age, gender, ethnicity, area deprivation, adverse hospitalization indicators	DKA and coma +ve correlated with DM prevalence
Weng 2000 [19]	England	332 DM (type I or II) pts	metabolic control, access to healthcare, clinical outcomes (neuropathy, retinopathy, proteinuria) and mortality	GIS mapping	age, gender, ethnicity, BMI, smoking, glycaemic control (HbA1c), Underprivileged Area Score	↑ morbidity and mortality in DM pts related to SES and ethnicity
Bocquier 2011 [16]	France	16 Marseilles cantons	prevalence estimation of treated DM	multilevel Poisson regression	area deprivation, population density, adjusted for individual-level factors (age, gender, low SES)	DM prevalence higher in more deprived and population-dense areas
Chaix 2011 [53]	France	2218 Paris census blocks	DM II prevalence, joint spatial correlation with study participation	multilevel logistic modelling	—	DM prevalence highest in areas with low educational attainment
Kravchenko 1996 [54]	Ukraine	27 admin regions	spatio-temporal estimation of DM I and DM II prevalence	regression, aggregate data	—	small area variation and general increase in prevalence over time

The statistical methods used in the 10 included papers are examined in detail in this section. Three studies used Bayesian generalized linear mixed modelling (GLMM), three used classic generalized linear modelling (GLM), one used classic GLMM, two used geographic information systems (GIS) mapping tools and one compared case provider ratios across regions. For Bayesian analyses involving Normal prior distributions, these were parametrized in terms of variances, unless otherwise specified.

### 3.1 Bayesian generalized linear models

#### 3.1.1 Multilevel logistic modelling

The paper by Chaix *et al.* used Bayesian multilevel logistic modelling to perform separate and joint modelling of neighbourhood determinants of both DM II prevalence and study participation, in 2218 census-block groups in the Paris metropolitan area [53]. Data from the RECORD Cohort Study were used. A GLMM was used to model the outcome of diagnosis with diabetes for each individual nested within their area of residence as a Bernoulli distribution with probability parameter *p*_*ik*_. The logit(*p*_*ik*_) was modelled as a linear function of individual and neighbourhood sociodemographic explanatory variables. Formulae for this multilevel logistic model are provided in the electronic supplementary material, appendix A.

The authors also performed coupled modelling of DM II and study participation, in order to identify selective participation related to neighbourhood, and account for potential bias on the associations with diabetes. A Markov chain Monte Carlo (MCMC) approach was used to simultaneously model study participation and diabetes prevalence using a Poisson model.

Separate modelling of DM II showed evidence that a low neighbourhood education was associated with higher odds of diabetes, controlling for individual education and self-reported financial strain. Joint modelling showed evidence that the odds of diabetes were slightly higher in high-participation areas.

#### 3.1.2 Sparse Poisson convolution conditional autoregression

Liese *et al.* used a variation of a Bayesian conditional autoregressive (CAR) and multivariate conditional autoregressive (MCAR) methods to separately evaluate geographical variation in DM I and DM II incidence, and to estimate joint spatial correlation between DM I and DM II, in youths aged 10–19 years in four US states [48]. Data from the *SEARCH for Diabetes in Youth Study* were used. CAR priors can be defined as a spatial structure in which the correlated random error of each region on a map of interest is described by a lattice of neighbouring regions, with *i*–*j* denoting that regions *i* and *j* are neighbours [45,55].

*Sparse Poisson convolution model.* A challenge with the data used in this study was the presence of sparse count data in some areas, violating assumptions of traditional Poisson models due to an excessive amount of zeros. The authors selected a sparse Poisson convolution (SPC) model to account for the sparseness of data. The SPC model is a variation of the CAR model with an added indicator variable denoting zero or non-zero count in any area. The SPC models used in this study to model DM I and DM II prevalence are described by the authors.

Joint spatial correlation between DM I and DM II was evaluated using a sparse Poisson MCAR model, which is a variation of the model with MCAR priors described by Gelfand & Vounatsu [56]. This type of model simultaneously models joint correlation between multiple outcomes while accounting for correlated error between spatial neighbours. In the model adapted by Liese *et al.* DM I and DM II were considered components of a vector of outcomes and a multivariate model applied. Joint spatial correlation between DM I and DM II was examined by calculating an empirical correlation between the RR estimates obtained for the SPC models using the Pearson correlation coefficient. Formulae for the SPC model and sparse Poisson MCAR model are provided in the electronic supplementary material, appendix A.

The study found evidence of geographical variation in DM I and DM II incidence, and evidence for joint spatial correlation between the two types of DM.

#### 3.1.3 Stratified generalized linear modelling

Congdon reported Bayesian GLM using MCMC in WINBUGS to estimate prevalence and mortality of clinically diagnosed diabetes (DM I and DM II combined) based on individual and neighbourhood sociodemographic factors in a small area prevalence study in England [52]. Data from 354 local authority areas from the Health Survey for England study were used. Furthermore, variations between areas in adverse hospitalisation indicators were compared to estimated diabetic prevalence rates. The authors considered three models, briefly described here.

In the first model, the stratified observed counts of diagnosed diabetes cases, stratified by gender, eighteen 5-year age bands and seven ethnic groups were modelled assuming a Poisson distribution.

Two GLMs were modelled, one with and one without age-ethnic group interactions. The coefficient for age was modelled using a random walk prior that assumes diabetes rates for successive age groups will tend to be similar [57].

In the second model, the prevalence gradient of diabetes (DM I and DM II combined) over neighbourhood deprivation quintiles was modelled using logistic regression. For each gender separately, the impact of age, ethnicity in four categories and neighbourhood deprivation quintile were assessed using a Bernoulli trial model for the presence of diabetes in each individual.

In the third model, diabetes mortality was modelled separately for each gender using Poisson regression. Formulae for the three models mentioned earlier are provided in the electronic supplementary material, appendix A.

The results showed evidence that diabetes prevalence rates varied from 2% to 5% across local authority areas. Male and female prevalence was comparable. For males, the area mortality rate from diabetes rose as area prevalence increased but was more regular over prevalence quintiles for females.

Further analysis was undertaken to assess health performance indicators of DM. Rankings were compared between 28 strategic health authority areas based on age-standardized rates of diabetic-ketoacidosis (DKA) coma and amputations, ratios of DKA coma episodes and amputation operations to estimated prevalent populations, and correlation of these indicators with prevalence rates. Results showed evidence that DKA and coma were positively correlated with prevalence, while diabetic amputation was not.

### 3.2 Classic generalized linear models and generalized linear mixed models

#### 3.2.1 Multilevel Poisson regression

Bocquier *et al.* used an age- and gender-adjusted multilevel Poisson GLM to model neighbourhood characteristics associated with prevalence of treated diabetes among beneficiaries, using data from drug reimbursement data from the General Health Insurance Scheme in southeastern France [16]. Individual gender and age characteristics were nested within geographical characteristics. Patients were classified as treated diabetic cases if they had oral antidiabetic medication or insulin dispensed three times or more within the year.

Results from this study found a crude prevalence of treated diabetes of 5.4%, and evidence that prevalence was significantly higher in more deprived and population-dense cantons independent of individual-level factors (age, gender, low socio-economic status (SES)).

#### 3.2.2 Generalized linear mixed modelling

Geraghty *et al.* used a GLMM approach to model HbA1c and low-density lipoprotein (LDL) cholesterol based on individual demographic and laboratory characteristics and neighbourhood SES quintile [49]. Registry data from 13 primary care clinics in Sacramento, California were used, and Euclidean distance from patients' homes to their primary care clinic calculated. GIS tools (ArcInfo) were used to analyse outcome disparities in a population of patients with DM II [58].

The first regression model assessed HbA1c level as a linear mixed model with random intercept and slope based on individual sociodemographic and laboratory characteristics, with practice characteristics or their primary care physician and clinic specialty as fixed effects and neighbourhood SES quintile as a random effect.

The second model dichotomized LDL cholesterol using a cutpoint of 100 mg dl^−1^, using mixed logistic regression. The same fixed and random effects were included as in the HbA1c model, with the addition of statin prescription. Formulae for both models are provided in the electronic supplementary material, appendix A.

Results of this study showed evidence of an association between neighbourhood SES quintile and HbA1c level. SES was not found to be associated with LDL control.

#### 3.2.3 Linear regression

Green *et al.* used analysis of variance and linear regression to identify sociodemographic, environmental and lifestyle factors associated with geographical variation in DM prevalence (DM I and DM II combined) in Winnipeg, Manitoba, using census, Health Epidemiology Unit, and hospital and physician claims data [17]. The authors used two methods to aggregate predictor and outcome data into high-risk areas for diabetes prevalence, firstly by aggregating to existing health administrative areas, and secondly by using a spatial scan statistic (SaTScan software [59]), both methods generating very similar results.

The spatial scan statistic places a circular window of varying size on a map surface, allowing its centre to move so that the window includes different sets of neighbouring areas at any given position and circle size. The window is placed alternatively at the centroid of each area and the radius varied continuously from zero, up to a maximum size including 50% of the population, using MCMC simulation to test for elevated risk of DM prevalence. The statistic assumes the number of cases in each geographical region to be Poisson distributed and tests the null hypothesis that within each age/gender group, the risk of DM is the same as in all regions combined [60].

With both aggregation methods, linear regression was used to model standardized DM prevalence as a function of socio-economic, environmental and lifestyle factors ecologically associated with the variability in prevalence, including self-reported Aboriginal status, education, income, family structure, unemployment, housing conditions, crime and smoking rates. Variables were log-transformed when necessary to meet assumptions of normality and homoscedascity (specific variables that were transformed were not given).

Results from this study showed evidence that higher DM prevalence was strongly associated with indicators of low SE status, poor environmental quality and poor lifestyles.

#### 3.2.4 Linear regression including temporal component

Kravchenko *et al.* [54] used mixed linear regression to separately model time trends in DM I and DM II prevalence, and the effect of smoking on prevalence of DM complications, in various administrative regions of the Ukraine. Statistical reports collected in 1990–1993 by specialized endocrinologic institutions were used in this study. For each region, the time trends for prevalence of DM I and DM II were separately interpolated by mixed linear regression. Formulae for this model are provided in the electronic supplementary material, appendix A.

Student's *t*-tests were used to test for differences in DM complications between two groups: smokers (10–30 cigarettes day^−1^ over 10 years) and non-smokers who had received preventative efforts for 5 years to correct nutrition, promote a healthy lifestyle, normalize body weight, metabolism and arterial pressure.

Results showed significant variation in the prevalence of DM II across regions, an overall increase in prevalence of both DM I and DM II over time, and evidence that prophylactic measures directed at a decrease in patient weight, the normalization of metabolism, arterial pressure and the elimination of pernicious habits promoted a decrease in diabetic complications.

### 3.3 Studies using geographic information system mapping

Weng *et al.* [19] investigated differences in metabolic control, access to healthcare, clinical outcomes (neuropathy, retinopathy, proteinuria) and mortality rates in a cohort of 610 diabetics living in different geographical areas of central London. Patients were clustered into prosperous, intermediate or deprived areas by electoral ward using Underprivileged Area Score (UPA score). GIS software (MapInfo) was used to analyse the geographical distribution of UPA of a sample of 332 patients [43].

Results showed evidence that patients in deprived areas were older, had higher BMI and worse glycaemic control than those in prosperous areas. Smoking was more prevalent in deprived areas. Prevalence of microvascular complications was related to geographical location, and age–gender-adjusted mortality rate was significantly higher in deprived than prosperous areas.

Noble *et al.* performed a feasibility study of geospatial mapping in Tower Hamlets, London, using ArcGIS, to examine 10 year risk of developing DM II as measured by QDScore [51,58,61]. Data from general practice electronic records on all non-diabetic individuals were used, and for each individual the QDScore instrument was used to calculate 10 year risk of developing DM II, and data were geocoded into areas.

Basic and smoothed visual maps were produced to identify areas of high and low 10 year risk. A ring map also visually illustrated availability of fast-food outlets, population density and percentage of green spaces for each area. A basic map of index of area multiple deprivation scores visually corresponded very closely with the basic map of 10 year DM risk.

The authors concluded that producing geospatial maps of DM risk from general practice electronic records was feasible and useful for public health and urban planning, but challenging due to data governance issues and technical challenges.

### 3.4 Study comparing case:provider ratio

#### 3.4.1 Mapping of child diabetes mellitus prevalence:paediatric endocrinologist ratio

Lee *et al.* [50] examined variations across US regions in the ratio of paediatric DM cases (types I and II, aged less than 18 years) to number of paediatric endocrinologists, and similarly, the ratio of obese children to paediatric endocrinologists. Data from the American Board of Pediatrics were used to estimate the number of board-certified paediatric endocrinologists by state, and data from the National Survey of Children's Health were used to estimate the number of children with diabetes and obesity by state.

Results showed evidence of geographical disparities in DM cases:endocrinologist supply, with a twofold difference between states.

## 4. Discussion

This review has shown that spatial studies have successfully been used to identify areas of high risk for DM II prevalence or incidence, to show disparities in case:provider ratio, metabolic control, access to healthcare, clinical outcomes and mortality rates across areas, and to compare temporal trends between areas. The two hierarchical models included, found evidence that spatial information influenced outcomes after adjusting for individual-level risk factors. Each included study found evidence for small area variation, and in addition, five found area-level indicators of lower SES, and two found area deprivation level to be positively correlated with outcomes, building on findings from several studies that find low area SES and deprivation to be risk factors for diabetes at an individual level [62–67]. One study found the clinical outcomes of ketoacidosis and coma to be positively correlated with area-level DM prevalence, indicating that areas of high prevalence are also prone to higher complication rates [52].

Joint modelling of multiple outcomes was shown to be useful in finding correlations between DM I and DM II, and between diagnosis with DM and study participation. These methods could usefully be extended to jointly model and examine correlations between incidence of DM II and DM II complications, or DM II and other chronic conditions, at either a mixed level (individual and area-level factors) or purely aggregate level. A full meta-analysis was not possible, owing to large differences in methodology, covariate inclusion and outcomes between the included studies.

In interpreting results and drawing conclusions from this review (step 8), a variety of area-level risk factors were confirmed by the included studies, the usefulness of including spatial information to describe geographical variation and identify regions of high excess risk was highlighted, and joint modelling of conditions was shown to be useful.

Three of the included studies used Bayesian methodology, which allowed several distinctive advantages over classical statistical methods. Individual effects nested within spatial effects were able to be described using hierarchical multilevel modelling and nested random effects [48,52,53]. Non-standard distributions were able to be fitted to these complex models, and the methods were suitable for situations with sparse or missing data. Different sources of uncertainty, such as spatial and non-spatial random error, were able to be incorporated and described within these types of models.

The study by Kravchenko *et al.* [54] included a temporal component in their model, allowing these authors to compare changes in DM prevalence over time between areas, identifying areas at higher risk for increases in prevalence. Information from spatio-temporal studies of this type may provide useful results influencing health policy and resource allocation decisions.

Standard linear models were used by Kravchenko *et al.* [54] to model DM prevalence as a function of year, and Geraghty *et al.* [49] to model HbA1c level as a function of multiple covariates. However, a GLM approach may be more appropriate in situations where the outcome variable is not normally distributed.

Our review methodology is based on the Cochrane collaboration guidelines for review methodology; however, the Cochrane model is implicitly based on the conventional medical model to exclusively identify patient-level risks from randomized clinical trials. Our review provides a template for a more holistic evidence review approach that also includes environmental risk factors, which is immediately applicable to other diseases.

The strengths of this systematic review include that Cochrane collaboration guidelines were followed in setting predefined aims, inclusion and exclusion criteria, selection of studies and assessment of bias. Selection of included studies was based on careful examination of modelling approaches used, and non-spatial studies were excluded. Transparent descriptions of models used in included studies are included in this review, with a view to outline methods that have been useful in examining spatial associations with DM II. Weaknesses of this review are that it does not account for bias from possible coverage and measurement errors, and that many of the included studies reported outcomes on both DM I and DM II, affecting the reliability of estimated outcomes purely for DM II. Other weaknesses are that an examination of reporting bias was not possible, and that there were insufficient studies similar enough to perform a meta-analysis.

## 5. Conclusion

Findings of this review show that incorporation of spatial information is useful and effective in modelling DM II and can identify spatial risk factors associated with DM II, and areas at high risk for DM outcomes and increasing DM burden over time. Several of the geographical risk factors associated with DM II outcomes, including green space, availability of healthy food, car-dominated transport and opportunities for exercise are amenable to modification. Bayesian methods allow joint modelling and examination of correlations between multiple outcomes. Although several spatial studies have been conducted examining DM II in the USA, UK and Europe, there is a lack of similar studies in other parts of the world. Spatial models conducted in these regions would be useful for identifying spatial risk factors associated with DM II and areas at high risk for DM outcomes, which would be beneficial in guiding public policy and management decisions.

## Supplementary Material

Appendix A: Formulae for spatial models included in systematic review
