# Supplementary material for: Spatial modelling of type II diabetes outcomes: a systematic review of approaches used
Source: R Soc Open Sci. 2015 Jun 17;2(6):140460. doi: 10.1098/rsos.140460 (PMC4632536; doi:10.1098/rsos.140460)
Supplement: Appendix A: Formulae for spatial models included in systematic review [file rsos140460supp1.docx]

**Appendix A: Formulae for spatial models included in systematic review**

3.1.1 Multilevel logistic modelling ([48](#_ENREF_48)):

A generalised linear mixed model (GLMM) was used to model the outcome of diagnosis with diabetes, $D_{ik}$, for each individual $i$ nested within their area of residence $k$, as a Bernoulli distribution with probability parameter $p_{ik}$. The logit($p_{ik}$) was modelled as a linear function of individual and neighbourhood sociodemographic explanatory variables, as follows:

$$D_{ik}\sim Bern(p_{ik})$$

$$logit(p_{ik})=X_{ik}^{T}\beta+U_{k}$$

$$U_{k}\sim N(\mu,\frac{1}{\tau})$$

where $X_{ik}$ is a vector of individual and area-level risk factors for individual $i$ within area $k$, $\beta$ is a vector of regression parameters, and $U_{k}$ is the uncorrelated random effect for each neighbourhood $k$, with mean $\mu$ and variance $\frac{1}{\tau}$ (an inverse of the precision $\tau$).

Priors distributions were specified for the precision and regression coefficients as follows: $\mu=0$, $\tau\sim Ga(0.5,0.0005)$, $N(0,10000)$ for each coefficient within vector $\beta$ with the exception of the intercept and coefficient for age, for which flat prior distributions were specified. A flat prior is a uniform prior distribution where the probability of any value within the distribution is a constant. The mean of the uncorrelated random effect was assumed known and set to zero.

3.1.2 Sparse Poisson Convolution conditional autoregression ([49](#_ENREF_49)):

For a lattice of neighbouring regions, with $i\sim j$ denoting that regions $i$ and $j$ are neighbours, the standard Poisson model using conditional autoregressive (CAR) priors for correlated random effect described by Besag et al. (1991) assuming a Poisson distribution for the observed number of cases $Y_{i}$in each area $i$, takes the following form:

$$Y_{i}\sim Po(E_{i}p_{i})$$

with offset $E_{i}$denoting the expected number of cases in area $i$, and $p_{i}$representing the relative risk of diabetes in area $i$.$Log(p_{i})$ is modelled as a linear equation as follows:

$$log(p_{i})=X_{i}^{T}\beta+U_{i}+V_{i}$$

where for area $i, X_{i}$ is a vector of area-level risk factors, $\beta$ is a vector of regression parameters, $U_{i}$ represents an uncorrelated random effect with no spatial structure, and $V_{i}$ represents a correlated random effect with CAR spatial structure.

The CAR prior assumes that the correlated random effect in area $i$, given the correlated random effect in area $j$, is given by:

$$V_{i}|V_{j}=v_{j},j\neq i \sim N\left( \mu\left( v_{j} \right),\frac{\sigma_{V}^{2}}{m_{i}} \right)$$

where $\mu\left( v_{j} \right)$ is average correlated random effect for the neighbours of area $i$, $m_{i}$ is the number of such neighbours, and $\sigma_{V}^{2}$ is the conditional variance of $V$. An advantage of using the CAR prior is that the conditional independencies can be modelled in MCMC estimation approaches, and allows spatial smoothing.

**Sparse Poisson Convolution model:**

The sparse Poisson convolution (SPC) models used in this study to model DM I and DM II prevalence are described by the authors as follows:

$$Y_{i}\sim Po(y_{i}\mu_{i})$$

$$\log\left( \mu_{i} \right)=\log\left( E_{i} \right)+\alpha\left( j \right)+u_{i}+v_{i}, j=1,2$$

where for census tract $i$, $y_{i}$ is the observed count, $E_{i}$ is the expected number of cases, $j$ is a binary factor indicating zero and non-zero observed counts (ie. $j=1$ if $y_{i}=0$ and $j=2$ if $y_{i}>0$, $\alpha(j)$ is a factored intercept for modelling zero and non-zero counts, $u_{i}$ is the uncorrelated random effect in and $v_{i}$ is the correlated random effect. The priors described for this model include a CAR prior for $v_{i}$ controlled by adjacent areas with common boundaries (first-order neighbours), intercept α$\sim N(0,1000)$, standard deviation of uncorrelated random error $\sigma_{u}\sim Unif(0,10)$ and standard deviation of correlated random error $\sigma_{v}\sim Unif(0,10)$.

**Sparse Poisson MCAR model:**

In the model adapted by Liese et al., DM I and DM II were considered components of a vector of outcomes and a multivariate model applied as follows:

$$Y_{i}\sim Po(Y_{i}\boldsymbol{\mu}_{i})$$

$$\log\left( \boldsymbol{\mu}_{i} \right)=\log\left( \boldsymbol{E}_{i} \right)+\boldsymbol{\alpha}\left( j \right)+\boldsymbol{U}_{i}+\boldsymbol{V}_{i}, j=1,2$$

where for census tract $i,$ $Y_{i}$ is a vector of multivariate observed counts for DM I and DM II following a Poisson distribution with mean $\boldsymbol{\mu}_{i}$,$\boldsymbol{\mu}_{i}$ is a vector of the means of the Poisson distribution of the multivariate health outcomes, $\boldsymbol{E}_{i}$ is a vector of the expected number of cases for multivariate outcomes, $\boldsymbol{\alpha}\mathbf{(}j)$ is a vector of factored intercepts for the multivariate outcomes where $j$ denotes the class of the observed count (zero or positive), $U_{i}$ is a vector of uncorrelated random effects and $V_{i}$ is a vector of correlated random effects.

Joint spatial correlation between DM I and DM II was examined by calculating an empirical correlation between the RR estimates obtained for the sparse Poisson convolution models using the Pearson correlation coefficient. The priors described for this model include a CAR model for $V_{i}$ controlled by adjacent areas with common boundaries, intercept α$\sim N(0,1000)$, standard deviation of uncorrelated random error $\sigma_{U}\sim Unif(0,10)$ and standard deviation of correlated random error $\sigma_{V}\sim Unif(0,10)$.

3.1.3 Stratified generalised linear modelling ([52](#_ENREF_52)):

The authors considered three models, briefly described here.

In the first model, the stratified observed counts, $Y_{ijk}$, of diagnosed diabetes cases, stratified by gender $i$, eighteen 5-year age bands $j$, and seven ethnic groups $k$, were modelled assuming a Poisson distribution, as follows:

$$Y_{ijk}\sim Po(E_{ijk}\mu_{ijk})$$

where $\mu_{ijk}$ models the impact of gender, age and ethinicity, and $E_{ijk}$ is the expected count in stratum ($ijk$). In this model, $E_{ijk}$ = $2.3*N_{ijk}$_,_ where 2.3 is the external standardisation rate of diabetic prevalence and $N_{ijk}$ is the population number in stratum $ijk$.

$log(\mu_{ijk}$)=${( X_{ijk})}^{T}\beta$

where ($X_{ijk}$) is a vector of area-level risk factors, and $\beta$ is a vector of regression parameters.

Two GLMs were modelled, one with and one without age-ethnic group interactions. The coefficient for age, $\beta_{j},$was modelled using a random walk prior that assumes diabetes rates for successive age groups will tend to be similar, as follows:

$\beta_{j}\sim N\left( \beta_{j-1},\frac{1}{Ƭ_{j}} \right)$ for $j=2,\ldots,18$

$\beta_{j=1}\sim N(0,1000)$ and precision $Ƭ_{j}$ $\sim Ga(1,1)$

The coefficients for gender, $\beta_{i}$, and ethnicity, $\beta_{k}$, were assigned fixed effect priors with corner constraints, $\beta_{i=1}$ =$\beta_{k=1}$ = 0,

$\beta_{i=2}\sim N(0,1000)$, $\beta_{k}\sim N\left( 0,1000 \right)$for$k=2,\ldots,7$

Additional priors used for the GLM with an age-ethnic group interaction term include:

$\beta_{jk}\sim N(0,\frac{1}{Ƭ_{jk}}$), where $Ƭ_{jk}\sim Ga(1,1)$. A sensitivity analysis was performed on the prior distribution for each precision, $Ƭ_{j}$and $Ƭ_{jk}$, with $Ga(1,0.1)$ and $Ga(1,0.001)$ priors also trialled.

In the second model, the prevalence gradient of diabetes (DM I and DM II combined) over neighbourhood deprivation quintiles m was modelled using logistic regression. For each gender separately, the impact of age ($j=1,\ldots,18$), ethnicity in four categories ($k=1,\ldots,4$) and neighbourhood deprivation quintile ($m=1,\ldots,5$) were assessed using a Bernoulli trial model for the presence of diabetes $D_{i}$ in each individual $i$, with probability parameter $p_{i}$_,_ as follows:

$$D_{i}\sim Bern(p_{i})$$

$$logit\left( p_{i} \right)=X_{i}^{T}\beta$$

where $X_{i}$ is a vector comprising ethnic group, age group and deprivation quintile for each individual $i$, and $\beta$ is a vector of regression parameters, including coefficients for $j,k,m$. Priors used were similar to Model 1, including a random walk prior on age categories $i$:

$\beta_{j}\sim N\left( \beta,\frac{1}{Ƭ_{j}} \right)$ for $j=2,\ldots,18$

$\beta_{j=1}\sim N$(0,1000) and precision $Ƭ_{j}$ $\sim Ga(1,1)$

The deprivation effects were assumed to follow truncated normal distributions, constraining sampling to produce a monotonic gradient as follows:

$\beta_{m}\sim N\left( 0,1000 \right)I{(\beta}_{m-1},\beta_{m+1})$, $m=2,3,4$

$$\beta_{m=1}\sim N\left( 0,1000 \right)I(-\infty,\beta_{m=2})$$

$$\beta_{m=5}\sim N\left( 0,1000 \right)I(\beta_{m=4},\infty)$$

where $I(a,b)$ is the interval from $a$ to $b$.

For ethnic group $k$, a normal prior with corner constraints was used:

$\beta_{k=1}$ = 0, $\beta_{k}\sim N\left( 0,1000 \right)$ for $k=2,3,4$

In the third model, diabetes mortality was modelled separately for each gender using Poisson regression. For males, let $Y_{i1}$ = observed deaths in area $i$, and $E_{i1}$=expected deaths in area $i$.

For females, let $Y_{i2}$ = observed deaths in area $i$, and $E_{i2}$=expected deaths in area $i$.

$Y_{i1}\sim Po(E_{i1}m_{i1})$; $\log\left( m_{i1} \right)=B(QM_{i})$

$Y_{i2}\sim Po(E_{i2}m_{i2})$; $\log\left( m_{i2} \right)=B(QF_{i})$

where $QM_{i}$ = male-prevalence quintile to which area $i$ belongs to and $QF_{i}$ = female-prevalence quintile to which area $i$ belongs to. A Poisson regression was assessed to be satisfactory for this model due to the absence of overdispersion.

**3.2 Classic generalised linear models and generalised linear mixed models:**

3.2.2 Generalised linear mixed modelling (GLMM) ([54](#_ENREF_54)):

The first regression model assessed HbA1c level as a linear mixed model with random intercept and slope based on individual sociodemographic and lab characteristics, with practice characteristics or their primary care physician and clinic specialty as fixed effects and neighbourhood SES quintile as a random effect:

$$H_{i}=X_{i}^{T}\beta+Z_{i}W_{i}+U_{i}$$

where $H_{i}$is the haemoglobin level for individual $i$, $X_{i}$ is a vector of risk factors with fixed effects for individual $i$, $\beta$ is a vector of fixed regression parameters, $Z_{i}$is a vector of risk factors with random effects for individual $i$, $Z_{i}$ is a vector of random regression parameters for individual $i$, and $U_{i}$ represents an uncorrelated random effect with no spatial structure.

The second model dichotomised LDL cholesterol using a cutpoint of 100mg/dL, using mixed logistic regression. The same fixed and random effects were included as in the HbA1c model, with the addition of statin prescription:

$$L_{i}\sim Bern(p_{i})$$

$logit\left( p_{i} \right)=$ $X_{i}^{T}\beta+Z_{i}W_{i}+U_{i}$

where for individual $i$, $L_{i}$ represents the outcome LDL>100, $p_{i}$ is the probability of having LDL>100, $X_{i}$ is a vector of risk factors with fixed effects, $\beta$ is a vector of fixed regression parameters, $Z_{i}$ is a vector of risk factors with random effects, $W_{i}$ is a vector of random regression parameters, and $U_{i}$ represents an uncorrelated random effect with no spatial structure.

3.2.4 Linear regression including temporal component ([58](#_ENREF_58)):

For each region, the time trends for prevalence of DM I and DM II were separately interpolated by mixed linear regression:

$$Y_{i}=a_{i}+\beta_{i}x_{i}+U_{i}$$

where for area $i,$ $Y_{i}$ is the estimated prevalence, $x_{i}$ is the year, $a_{i}$ is the intercept, $\beta_{i}$ is the coefficient for year, and $U_{i}$ is an uncorrelated random effect with no spatial structure.
